# Public health education: A report from Mosul and a plan for change

**DOI:** 10.1186/1471-2458-5-29

**Published:** 2005-03-31

**Authors:** Gail D Hughes, Ally Mack, Kathie Stromile Golden

**Affiliations:** 1 Department of Preventive Medicine-Epidemiology, University of Mississippi Medical Center, 2500 North State Street, Jackson, MS 39216, USA; 2 Mississippi Consortium for International Development (MCID), 1225 Robinson Street, Jackson, MS 39203, USA; 3 Department of International Programs, Mississippi Valley State University, 14000 Highway 82 W., #5098 Itta Bena, MS 38941-1400, USA

## Abstract

**Background:**

Today Iraq suffers from severe shortages of food, medicine, clean water and adequate sanitation. Malnutrition and communicable diseases are major factors in the rising morbidity and mortality rates. However, supplies and equipment are insufficient or outmoded, and public health training is outdated. The Universities have been unable to help because under-funding and isolation from their professional colleagues has limited their effectiveness.

**Methods:**

To revitalize public health education, we describe a partnership between a US education consortium and the University of Mosul that will be carried out in the next several years. The plan is based on "three R's": Recovery from the past damage due to war and neglect; Retooling of key public health faculty to remedy the years of isolation and restriction of activity; and Reestablishment of the University as a resource for the its constituents, for the community and for other educational institutions. In all these activities, Iraqi minorities, especially women, will participate and contribute.

**Conclusion:**

The work to repair the public health educational infrastructure has just begun. When completed, it will represent a small but necessary step in restoring normalcy to the people of Mosul, and of Iraq.

## Background

### The current situation in Iraq

Today Iraq is a country plagued by poverty, deprivation, and disease. The war with Iran in the 1980's, the Persian Gulf War in 1991, a crippling economic blockade with lasting economic sanctions, and finally the recent war to oust the Hussein regime from power have caused severe shortages of food, medicine, clean water and adequate sanitation. The current state of the health system has been described as "a shambles" (Aziz, 2003).

The problems are both acute and chronic. Malnutrition, high rates of communicable diseases, and increased mortality have been documented by Iraqi scholars and international researchers. Children, who comprise nearly half the Iraqi population (UNICEF, 2003. At a glance: Iraq) are most vulnerable and have been most studied. Jawadi found that 38 percent of 1881 children ranging in age from newborn to five who attended a Mosul healthcare center were chronically malnourished and 30 percent suffered wasting attributable to "the increase of water-borne diseases like severe gastroenteritis together with the shortage of basic food" (Al-Jawadi, 1996). A later investigation reported increased mortality as the result (Al-Jawadi, 1999). UNICEF studies confirm these findings in the country as a whole (UNICEF, 2003. The Situation of Children) and chart the rise in mortality risk for small children over the last 14 years (Ali MM 2003). Beginning in 1974, mortality rates for infants and for children under five in Iraq declined overall until 1990, but then essentially doubled and remained high. Now one in every eight children will die before age five, making Iraq's rate the 36^th ^highest of about 200 countries reporting to UNICEF. (UNICEF, 2003. The state of the world's children pp 102-5)

The shortage of food in Mosul reported by Jawadi is experienced throughout Iraq, contributing to the risk of vulnerable populations. In May 2003, UNICEF reported that acute malnutrition among children had almost doubled since before the war, and stood at 7.7 percent. Part of the cause was the collapse of the Primary Health Care Centres, which identify and treat malnutrition (UNICEF, 2003. Restore public health system for malnourished children). Of the country's 1200 Centres, some 20 percent have been destroyed or looted, while half the balance needs extensive repairs. Almost none have a consistent water supply, and about a third have no physician on staff (Volker 2004).

Poor sanitation, polluted water and decline in health services are among the reasons for this disastrous situation, (Al Nouri 2003) exacerbated by the recent military hostilities. Prior to the first Gulf war, almost all the urban population of Iraq had piped, filtered and disinfected water (Martone 2003). Destruction of the electric and the supply infrastructure since then, coupled with neglect, has resulted in severe water pollution. Concurrently, communicable disease rates have risen to near-epidemic proportions, with 70 percent of children in Baghdad suffering from diarrhea at the end of the present war (Martone 2003). Shortages of certain laboratory agents and reagents that could not be imported during the sanctions are another major factor in the increased morbidity and mortality from meningitis, influenza, pneumonia, tuberculosis, and other infectious diseases. Even now, laboratory and medical supplies are extremely difficult to get, and the staff of one hospital in Mosul fought off looters of these items with their bare hands (Dyer, 2003). The dearth of equipment and the outdated training of public health professionals contribute to the difficulty of making an accurate diagnosis. For example, one study found that the antiquated methods and technologies of diagnosis were responsible for a 35 percent misdiagnosis rate in cases of typhoid fever (Al-Dabbagh, 1999).

Iraq's health system was once among the most advanced in the Middle East (Aziz 2003). There are plans for rebuilding Iraqi science (Stone 2004), and public health training has been listed as a priority in this effort by the Iraqi Ministry of Health and others (Fleck 2003). Because progress depends on professionals who can lead the way forward by training others, developing a cadre of skilled workers is essential for the task of rebuilding health care. However, virtually no training of health professionals has been available for 15 years, medical libraries are out of date, Internet access is minimal, and educational facilities have been destroyed. (Amin 2003; Furber 2004). Thus the training must begin at the top, with those who will become trainers themselves.

The need for improved professional training runs through all proposals for Iraqi reconstruction. In particular, public health training is a priority because many of Iraq's health problems can be addressed with public health solutions. Here we describe a plan at the University of Mosul that is now being implemented, with the goal of reestablishing a major Iraqi university as a source of expertise, education and service in public health.

## Methods

### A plan to reestablish the University of Mosul: The three R's

The Mississippi Consortium for International Development (MCID) is a collaborative endeavor of four historically black institutions of higher learning in Mississippi (Alcorn State University, Jackson State University, Mississippi Valley State University, and Tougaloo College). The Consortium provides international human resource development training and technical assistance and currently maintains formal partnerships with more than 50 universities worldwide. In July 2003, MCID established an International Partnership between the Consortium members, the University of Mississippi Medical Center and the University of Mosul to plan for the revitalization of this formerly eminent university, and a plan was mapped out for the revitalization in the next six months. Public health and sanitation were chosen as the focus of the effort because they are essential areas in Iraq's recovery. The plan, known as the HEAD (Higher Education and Development) Project, is now in its first year of operation.

Three phases of the plan, the "Three R's", describe the thrust of the HEAD Project:

• Recovery from damage at the University of Mosul due to political party policies, looting, and allowing a return to normal functioning.

• Retooling of key faculties, updating knowledge to allow their reentry into professional activities worldwide.

• Reestablishment of the institutional viability of the University, as well as its relationships with community organizations and other institutions of higher education.

The Partners in the HEAD Project support full participation of Iraq's minorities, including women, in the implementation of the three R's. This policy of inclusion gives the University a leadership role in the development of the new Iraq.

#### Recovery of the facility

The most immediate need of the University of Mosul is replacing laboratory, classroom and office equipment, and textbooks destroyed or stolen during the April 9–10 2003 looting rampage. The University has initiated repairs to physical facilities, but decisions facing the University go far beyond mere replacement of a basic stock of equipment and supplies. More than two decades of under-funding and nearly complete isolation from the outside world since 1991 have rendered the Mosul faculty unable to specify their needs. When asked, professors readily admit that they are not aware of the advances made in their academic disciplines or of the options available to them. They suggest that the best way to solve the dilemma is to include equipment identification as a part of the Partnership faculty exchange program (described below). To rush these decisions could result in purchasing out of date, inappropriate, or unneeded equipment.

The HEAD Project proposes to fund limited equipment purchases throughout the University's colleges rather than exclusively for the Colleges of Medicine, Nursing, and Engineering, where public health training is usually carried out. Project staff believes that the viability of individual colleges will depend upon the institution as a whole being functional. University officials, with MCID, are determining which purchases should be made immediately to assure that essential services are available to the administration, faculty and students to conduct classes and support sensitive research projects.

In the past, the University of Mosul has benefited from donations of equipment, textbooks and journals from U.S. companies and private individuals. MCID proposes to conduct a campaign during the initial year to elicit additional support for the HEAD Project from a select number of private entities at the beginning of the project. The HEAD Project will target at least 15 U.S. and foreign businesses operating in Iraq with established corporate social responsibility funding programs. The next step is an intensive round of meetings with the Baghdad-based representatives of these companies to identify activities within the HEAD Project that match their corporate interests or the priorities of their social responsibility programs. Past experience suggest that the matching process does not take long but the decision can take several months. Obtaining social responsibility funds invariably involves approval by the corporate headquarters, even though the recommendation of their in-country personnel is typically followed. In the interim, and in addition to these public-private partnerships described above, additional donors of smaller gifts such as back issues of technical journals and excess laboratory equipment will be sought.

#### Retooling the faculty

There is broad consensus among the more than 2,000 University of Mosul faculty members that the intellectual isolation (some would argue 25 years) has left them woefully out of touch with their professions. They argue convincingly that to deliver a quality education to their students and to participate in the debates that define the outer reaches of their disciplines, a program must be undertaken to rapidly bring them up to date. A critical first step is improved access to information via the Internet and subscriptions to academic journals. Beyond that, however, direct contact with colleagues in the Western world is essential, through refresher courses, faculty exchange programs, sabbaticals, and participation in international conferences. The majority of exchange programs will bring faculty to the United States, but visitations by MCID faculty to assist in designing the retooling agenda are also planned if security permits.

Retooling cannot be completed quickly, for the teaching pool must be maintained at an adequate level during the academic year. Therefore, we propose to spread this component over a three-year period. The cadence will be negotiated by a coordinating committee that includes representatives of the Colleges of Engineering, Nursing and Medicine, where most of the training will take place, with the President of the University serving as the final arbiter of the distribution of the training programs among the colleges.

The first year of work requires achievable objectives while simultaneously focusing on long term capacity building. Several key efforts are now ongoing or planned for the near future. These are set out below.

***Faculty refresher courses*** address the request of University of Mosul professors for two-week reviews of key public health areas. Courses for senior staff include clinical epidemiology, primary health care, demography, and maternal and child health. Recommended refresher courses for junior staff are biostatistics, social and behavioral sciences, and occupational health. The courses are based on the model, Train the Trainer, whereby the newly trained faculty at the University of Mosul will replicate the courses for students and offer them to other public health professionals and medical practitioners.

***Faculty Exchange Programs**** allow *faculty to learn first-hand of innovative and state-of-the-art technology and trends in their respective professions by visiting MCID institutions. The major exchanges may be for any period from two weeks to six months. Faculty are surveyed on their research interests and needed areas of growth; they are then matched to an MCID institution and faculty member to assist them in professional development. Collaborative research, curriculum development, and participation in lectures, grand rounds, and national and international conferences are all encouraged. In addition, a Research Mentoring Program, which will foster collaboration between faculty at Mosul University and MCID faculty/research investigators, is planned in order to provide increased one-to-one faculty interaction.

An MCID ***faculty residency*** will target the development of a specific project. For example, Mosul faculty specializing in environmental health might take residency at Jackson State University and participate in work addressing a specific environmental health project being studied there; similarly, an MCID faculty member would assume residency at the University of Mosul to serve as technical advisor in the development of targeted activities. The faculty residencies are one to four months in length.

***Conferences and workshops*** are essential components of faculty retooling. Funds are available to support participation in national and international activities in areas of public health, sanitation and nursing. Examples are:

• Identification of best practices and applications in specific areas of public health, sanitation and nursing given the needs of northern Iraq;

• Participation in skills-building workshops in targeted areas of professional growth in public health, sanitation and nursing;

• Exchange of research findings and technical information between the University of Mosul and other higher educational institutions in Iraq and the Middle East, and/or Iraqi governmental structures in public health, sanitation and nursing.

• Representation of the University of Mosul and/or Iraq at national and international conferences in public health, sanitation and nursing.

All of the aforementioned programs have an emphasis on minorities, especially women. Furthermore, allowances will be made to ensure active participation of minority faculty. In addition, research and course development on health issues specific to minorities will be strongly encouraged through each initiative.

#### Reestablishment of institutional viability

No institution, especially an educational institution, can thrive in isolation. Frustrated by years of financial neglect, over-crowding, and deprived of contact with the outside world, the faculty and staff of the University of Mosul have remained at their institution only out of sheer dedication. Over the past 12 years, many of their colleagues have left for more receptive environments where they could teach and conduct research in tranquility. For those who remained at the University, life has been difficult. They endured policies intended to exclude the interests of ethnic and religious minorities; were frustrated in their attempts to communicate with their professional colleagues by all means including the postal service and the Internet; and their every move was closely scrutinized to detect evidence of disloyalty. One of the first steps to restore the University of Mosul's viability must be reconnecting faculty and students with peers and colleagues inside and outside Iraq and reestablishing the University's outreach to community-based organizations. The HEAD Project is accomplishing this via several avenues.

#### Expansion of internet access to the administration, faculty and students

Internet access expansion can be organized in a variety of ways, from the creation of a centralized facility to one that is totally decentralized, with a mixture of the two also possible. Engineers prefer a college-based system that would be integrated into the specialized computer system in place in their College. Because of the need to accomodate sensors and other devices specifically designed for engineering functions, a centralized system would not serve their needs. A plan for upgrading Internet access can only be constructed after a user needs survey, development of alternate plans, and a study of their budgetary implications.

Information technology is necessary for national and international communications, in addition to staying current on public health and medical advances. While computer equipment, servers and Internet connectivity are being restored, a University of Mosul/MCID website is being developed which will allow for national and international faculty exchanges, the E-book program (see below), and other informational services. As time progresses, the web site will be expanded to link all participating institutions. The importance of an adequate Internet network and universal access cannot be overestimated in a country where postal and telephone services are primitive, at best.

***The electronic book program*** is an exciting option in the search for access to books, now extremely limited in Iraq. Electronic books represent a vast social opportunity to increase access to information, books, and education, especially for disadvantaged communities. For example, with collections of cultural texts captured and offered in an easily accessible format, the histories of Iraq's diverse communities can be preserved and shared, fostering awareness and understanding of the distinct heritage of each ethnic and religious group. The economics of electronic collection delivery are powerful; the incremental cost to deliver another book as an electronic text file is extraordinarily low. A repository of scanned and publisher-supplied electronic books, as well as thousands of public domain and freely available books, is being established on a server at the university, and these ebooks will be made available to students, faculty and professionals in all of Iraq. Electronic access will be free at the university, and books printed on demand will be available at prices closely linked to local production costs.

***Community outreach programs*** help the university reach out to the community it serves. The HEAD Project proposes to finance a variety of outreach programs including for support of work of civil society organizations, those working in public health and human rights, faculty research programs and student thesis research, and a mini-grants program to encourage academic inquiry dedicated to addressing the specific needs of disenfranchised minority groups, including Iraqi women.

**Mini-grants** funded by the HEAD Project will provide funds for several short-term projects. Using a review process similar to that of national and international agencies, proposals will be peer reviewed and granted expeditiously to ensure timely completion. Funded projects may include collaborations with other colleges on the University of Mosul campus, other Iraq universities, and the MCID affiliates. They may supplement activities in other HEAD Project programs. For student theses (master's program), emphasis will be placed on those research projects that promote and focus on outreach in their communities.

**Training for Research Awareness in Nursing (TRAIN)** is a program designed to increase the pool of nurse researchers by providing theoretical and experiential learning activities related to the research process, as well as nursing input into the health of the community and further post-graduate preparation. Academic development will be achieved in a nine-week program based on a successful US model by 1) providing intensive theoretical and practical experience with research related to racial/ethnic and gender-specific (female) health issues and concerns; 2) acquainting the student with materials and techniques necessary to facilitate entry into graduate programs such as nursing and public health, and 3) facilitating networking with faculty and students of MCID. This allows for inter-university relations between the School of Nursing and College of Medicine, for example, and it can be a pilot program for other Iraqi institutions as well as collaborations within institutional departments.

The **community sustainable health outreach program (CSHOP)** is proposed for the University of Mosul's Department of Community Medicine. The program, with key faculty from the Colleges of Engineering, Nursing and Medicine, will have as its goal the development of sustainable health outreach programs in sanitation. These include ensuring proper water safety (teaching techniques of hand washing, boiling water, for example), preventive health practices (proper nutrition, breastfeeding, immunizations) and educational awareness (media campaigns, religious center outreach, informational health materials). The faculty team will consist of representatives from each college with technical expertise in selected areas of public health and sanitation.

Existing NGO's will be enlisted as an immediate resource and mechanism for the University of Mosul's CSHOP projects. In addition, key informants and expert opinions will be acquired from identified "gate keepers" in the community, with the goal of developing new NGOs to provide outreach and services to various targeted communities (women, ethnic/racial minorities). The CSHOP will conduct needs assessments to determine current community health concerns and provide technical assistance/consulting, professional development workshops, health classes, health screenings and preventive services, and limited funding for outreach activities. The CSHOP advisory board will include representatives from each NGO and designated community gatekeeper and will meet on a regular basis so that communication between the University and community remains open and fluid as community health needs are met. The CSHOP personnel will consist of both graduate and professional students (medical, nursing) supervised by faculty mentors. This service will staff the CSHOP as well as serve as a training experience for students during their academic pursuits.

#### Expansion of inter- and intra-university relations

Finally, the HEAD Project Partners intend to support cross-university programs in public health, nursing and sanitation between the University of Mosul and other Iraqi institutions of higher education in other regions. The strengthening of inter-university ties will help reduce the isolation each university has suffered over the past decade. At the same time, MCID institutions in the US will work toward the establishment of joint degree programs with the University of Mosul in public health, nursing, and sanitation, which could include joint research, student study abroad, and long distance learning. Planning for these programs is underway in this initial year of the HEAD Project. Finally, Jackson State University intends to propose a Masters of Public Health joint degree program with the University of Mosul, once faculty assessments have been initiated and interest in the program fully determined.

## Conclusion

The decline in public health services in Iraq has contributed to a rise in Iraqi morbidity and mortality. Severe acute and chronic problems need to be addressed. The universities have been unable to help in training public health personnel. Now, with the change of regime in Iraq, an important opportunity exists to chart a new course. We propose an International Partnership between a US educational consortium and the University of Mosul in northern Iraq consisting of three essential parts: recovery from past damage with return to normal university function, retooling key faculties after years of isolation, and reestablishment of the institutional viability of the University of Mosul. This resurgence of public health education is a small step, but a necessary one in restoring normalcy to the people of Mosul, and of Iraq.

## Competing interests

The author(s) declare that they have no competing interests.

## Authors' contributions

GDH and AM were involved in the design and concept of project, and were awarded the funding for the research. All authors were involved in the acquisition of results and drafting of the manuscript. All authors read and approved the final manuscript.

## Pre-publication history

The pre-publication history for this paper can be accessed here:


